
JOURNAL INFORMATION
==============================
NLM Title Abbreviation: ScientificWorldJournal
Journal ID: ScientificWorldJournal
NLM Journal ID: 101131163
Journal ID: TSWJ

The Scientific World Journal

ISSN: 2356-6140
EISSN: 1537-744X
Publisher: Wiley

ARTICLE INFORMATION
==============================
PMCID: PMC6083840
PMID: 30147536
DOI: 10.1100/tsw.2001.109
Article ID: 179093
Article version: 1
Subjects: Short Report

The Role of the MRG Gene Family in Replicative Senescence and Immortalization

Leung James K.
Pardo Patricia
Pereira-Smith Olivia M. *
Huffington Center on Aging, Baylor College of Medicine, One Baylor Plaza, M320, Houston, TX 77030, USA
*Olivia M. Pereira-Smith: osmith@bcm.tmc.edu
Publication date: 2001
Electronic publication date: 2001 Feb 2
Volume: 1
First page: 66
Last page: 66
Copyright: Copyright © 2001 James K. Leung et al.
Copyright year: 2001
License: This is an open access article distributed under the Creative Commons Attribution License, which permits unrestricted use, distribution, and reproduction in any medium, provided the original work is properly cited.
License URL: https://creativecommons.org/licenses/by/3.0/

Funding: Doris and Curtis Hankamer Foundation National Institute on Aging R37AG05333 National Institute on Aging POIAG13663

==============================
Miami *Nature Biotechnology* Short Reports
*TheScientificWorld* (2001) 1(S3), 66SR
ISSN 1532-2246; DOI 10.1100/TSW.2001.109

### THE ROLE OF THE MRG GENE FAMILY IN REPLICATIVE SENESCENCE AND IMMORTALIZATION

James K. Leung, Patricia Pardo and Olivia M. Pereira-Smith*

Huffington Center on Aging, Baylor College of Medicine, One Baylor Plaza, M320, Houston, TX 77030, USA
*osmith@bcm.tmc.edu

**INTRODUCTION.** Replicative senescence, the terminal loss of proliferation that normal cells undergo in vitro, is considered a model for aging at the cellular level. It is also considered a mechanism of tumor suppression because fusion of normal with immortal, tumor derived cell lines yields hybrids that regain growth control and cease division. Additional genetic analysis has revealed that a large number of immortal human cell lines assign to only four complementation groups for indefinite division (A-D) and microcell fusion studies have identified human chromosomes 1,4, and 7 as carrying the cell senescence gene loci for groups C, B and D respectively. We have cloned a gene on chromosome 4, MORF 4, that following transfection induces senescence only in immortal cell lines assigned to group B. MORF 4 is a member of a gene family MORF related genes (MRG) and the three expressed family members share interesting common motifs, including a leucine zipper region, a helix-loop-helix domain and nuclear localization signal. MRG 15 has a unique chromodomain at the amino terminus, MORF 4 has lost this region and MRG X has another unique amino terminal sequence with no known homology. We here present an update on our studies with these genes.

**METHODS.** To identify proteins that interact with the MORF 4, MRG 15 and MRG X proteins we used the techniques of yeast two-hybrid, GST-pull down/Western, and immunoprecipitation (IP)/western analyses. Fractionation of nucleoprotein complexes was done on sucrose gradients. Transfection was performed using lipofectamine plus according to manufacturers instructions.

**RESULTS.** We have identified a novel protein PAM (protein associated with MRG) that interacts with the MRG proteins. This identification was initially done by yeast two-hybrid analysis and confirmed by GST- pull-down and IP/Western experiments. We have also determined that MRG 15 and MRG X are in multiple nucleoprotein complexes. They co-sediment on sucrose gradients with PAM and are present in the same immunoprecipitates. We are currently determining whether they are present in the same complex(es) or whether they interact with PAM in an independent complex(es), since yeast two-hybrid studies indicate they do not interact directly with each other. Both MRG X and MRG 15 can activate a promoter-reporter construct of the b-myb gene and we are characterizing the region(s) of the proteins that is involved by the use of deletion mutants. We are also attempting to characterize the other components of the complex(es).

**DISCUSSION.** Our studies with MRG X and MRG 15 indicate these proteins will be present in complexes that activate genes, and most likely be important in cell cycle progression. Since

MORF 4 is a truncated version of MRG 15 that lacks the N-terminal chromodomain, we hypothesize that during induction of senescence in group B cell lines, it displaces MRG 15 and MRG X from their complexes and disrupts cell division. Indeed, confocal microscopy results suggest that this occurs. However, additional information is needed to support this hypothesis.

**ACKNOWLEDGEMENTS.** This work was supported by the Doris and Curtis Hankamer Foundation and NIA grants R37AG05333 and POIAG13663.

**REFERENCES.**

1. Bertram, M.J., Berube, N.G., Hang-Swanson, X., Ran, Q., Leung, J.K., Bryce, S., Spurgers, K., Bick, R.J., Ning, Y., Clark, L.J., Parkinson, E.K., Barrett, J.C., Smith, J.R., and Pereira-Smith, O.M. (1999) Mol. Cell. Biol. 19, 1479-1485
